# Association between a novel obesity index WWI and chronic kidney disease in a Chinese middle-aged and older population: a national prospective cohort study

**DOI:** 10.1038/s41598-026-48320-w

**Published:** 2026-04-18

**Authors:** Juan Xie, Haijing Dou, Hongmei Wang, Meng Su, Yi Lei, Lin Bai, Jun Liu, Hailun Li, Yong Xu, Xiang Li, Donghui Zheng

**Affiliations:** 1 Department of Nephrology, The Affiliated Huai’an Hospital of Xuzhou Medical University and Huai’an Second People’s Hospital, Huai’an, China; 2 Department of Clinical Laboratory, The Affiliated Huai’an Hospital of Xuzhou Medical University and Huai’an Second People’s Hospital, Huai’an, China; 3 Huai’an Key Laboratory of Chronic Kidney Disease, The Affiliated Huai’an Hospital of Xuzhou Medical University and Huai’an Second People’s Hospital, Huai’an, China

**Keywords:** Weight-adjusted waist circumference index (WWI), Chronic kidney disease, Obesity, Prospective study, Nephrology, Risk factors

## Abstract

Obesity is a significant risk factor for chronic kidney disease (CKD), but traditional body mass index (BMI) cannot distinguish between muscle and fat mass. Weight-adjusted waist index (WWI), as a novel obesity indicator, better reflects central adiposity and body composition; however, its longitudinal association with CKD incidence remains unclear, particularly regarding sex differences. This study utilized prospective cohort data including middle-aged and older adults with a median follow-up of 8.0 years. WWI was categorized into sex-specific quartiles. Prior to covariate adjustment, the log-rank test and Kaplan-Meier curves were used to assess cumulative risk of CKD incidence across WWI groups. Cox proportional hazards models were employed to evaluate the association between WWI and CKD risk. CKD was defined as estimated glomerular filtration rate < 60 mL/min/1.73 m² or self-reported kidney disease during follow-up. Results showed that WWI was significantly associated with CKD incidence in females, with hazard ratios of 1.56 (95%CI 1.04–2.34), 1.59 (95%CI 1.08–2.34), and 1.53 (95%CI 1.01–2.31) for Q1, Q3, and Q4, respectively, compared with Q2, whereas no significant association was observed in males. ROC curve analysis demonstrated that WWI (AUC = 0.80) exhibited superior predictive performance for CKD in females compared with traditional obesity indices BMI (AUC = 0.78) and WC (AUC = 0.79) (*P* < 0.05). Sensitivity analyses confirmed the robustness of these findings. This study suggests that WWI is an independent predictor of CKD risk in middle-aged and older women, providing a novel anthropometric indicator for renal disease prevention and control in female populations.

**Supplementary Information:**

The online version contains supplementary material available at 10.1038/s41598-026-48320-w.

## Introduction

Chronic kidney disease (CKD) is a chronic progressive disorder that affects 820 million individuals globally and is projected to become the 5th leading cause of mortality by 2040^1^. Despite the emergence of novel therapeutic drug classes, such as sodium-glucose cotransporter 2 inhibitors, glucagon-like peptide-1 receptor agonists, and budesonide, that have expanded the treatment armamentarium for CKD, a significant proportion of patients still progress to end-stage renal disease (ESRD). If poorly managed, these patients require renal replacement therapy, including hemodialysis, peritoneal dialysis, or kidney transplantation^2^. CKD has a poor prognosis, causing heavy mental and economic burdens to individuals, families, and even society^3^. Due to the high incidence and severe consequences of CKD, there is a need for early recognition and a better understanding of its risk factors.

Obesity is increasingly being recognized as a disease, not simply a physical phenotype. This global challenge has experienced a dramatic increase in prevalence worldwide. According to a 2024 study in The Lancet, global obesity prevalence in children and adolescents quadrupled from 1990 to 2022. Even more concerning, adult female obesity rates have more than doubled, while male rates have nearly tripled^4^. In China, the prevalence of obesity is also increasing rapidly, with approximately 46% of the adult population being classified as overweight or obese^5^. In a large cohort study of 320,252 adults, Hsu et al. identified overweight and obesity as strong, potentially modifiable risk factors for the development of end-stage renal disease (ESRD)^6^.Accumulating evidence indicates that adipose tissue distribution patterns, rather than BMI-defined obesity per se, are mechanistically linked to diverse metabolic aberrations including hyperinsulinemia, hypertension, dyslipidemia, and atherosclerosis. Therefore, measures of visceral adiposity, such as elevated waist circumference, demonstrated superior predictive value for CKD compared to body mass index^7,8^.

Previous population-based studies have mainly used body mass index (BMI) and waist circumference (WC) as indicators of obesity^9^. However, these indicators cannot distinguish between fat and muscle composition. WC constitutes a simple yet robust anthropometric indicator of abdominal adiposity, reflecting visceral obesity more accurately than BMI^10^. Despite capturing regional fat distribution, WC exhibits a substantial correlation with BMI. Recent evidence indicates that weight-adjusted waist index (WWI), derived by standardizing the WC–weight relationship, enables precise obesity assessment, integrates the advantages of WC while attenuating its correlation with BMI, and represents a novel anthropometric indicator of fat and muscle composition^11–13^. The WWI is a novel obesity index that standardizes waist circumference and weight by dividing waist circumference (cm) by the square root of body weight (kg), highlighting the strength of waist circumference while decreasing the relationship with BMI^14^.

However, few cross-sectional studies have confirmed the positive correlation between the WWI and CKD^15,16^. To address this gap, this study was designed to prospectively examine the longitudinal association between graded WWI levels and incident CKD, thereby furnishing objective scientific evidence for risk factor characterization, early intervention, and preventive strategies.

## Methods

### Population and study design

This study is based on the Chinese Health and Longevity Longitudinal Study (CHARLS), an ongoing dynamic cohort study that began in 2011 and randomly selected community residents aged over 45 years from 22 provinces in China. The baseline survey covered 150 regions and 450 villages/urban communities, indicating the overall situation of middle-aged and older population people in China. Follow-up waves were conducted in 2013 (Wave 1), 2015 (Wave 2), 2018 (Wave 3) and 2020(Wave 4). A comprehensive description of the study and its corresponding data is available at https://charls.charlsdata.com/users/sign_in/zh-cn.

**Fig. 1:**
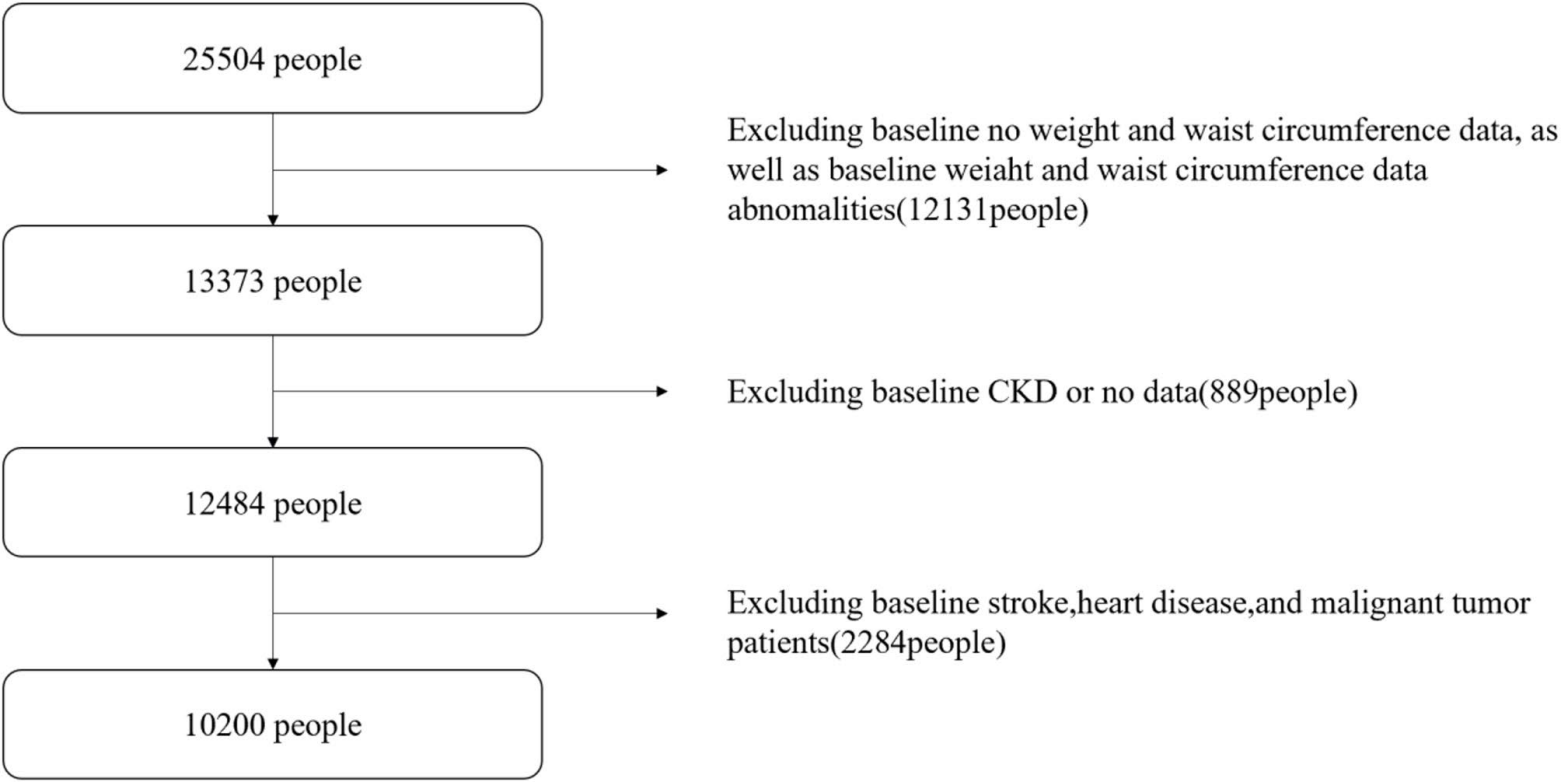
Flowchart of the study population.

This study enrolled 25,504 patients. After implementing the exclusion criteria, i.e., (1) no weight or waist circumference data or baseline weight or waist circumference data abnormalities, (2) previous chronic kidney disease or no chronic kidney disease data, and (3) stroke, heart disease, or malignant tumor status, 10,200 patients were eventually recruited into the trial (Fig. 1).

### Data collection and variable definitions

The WWI (cm/√kg) was computed by dividing WC (cm) by the square root of weight (kg)^11^. Together with the measures of WC and height included in the manual, a vertical stadiometer was utilized to measure height with a precision of 0.1 cm. WC was assessed horizontally at the umbilical level and recorded to the nearest 0.1 cm. The WWI was designated as the exposure variable in this analysis.

The BMI was computed by weight (kg)/height^2^ (m).

### Definition of CKD

CKD is defined as abnormalities of kidney structure or function that persist for at least three months and have implications for health^17^. CKD was based on self-reported physician diagnoses (‘Have you ever been told by a doctor that you have kidney disease?’) or personal eGFR levels from Wave 2 to Wave 4. If an affirmative answer was given by an individual or his/her proxy respondent or eGFR < 60 mL/min per 1.73 m^2^, then the individual was categorized as experiencing first-time CKD in his/her life (except for tumor or cancer). The eGFR was calculated using the Chronic Kidney Disease Epidemiology Collaboration creatinine equation with an adjusted coefficient of 1.1 for the Chinese population^18^. CKD was considered an outcome variable.

### Covariates

Furthermore, a set of covariates was incorporated into the analysis, including age, sex, ethnicity, education level, smoking status, medical history of diabetes mellitus, hypertension, and hyperlipidemia, as well as laboratory parameters such as triglycerides, high-density lipoprotein cholesterol (HDL-C), low-density lipoprotein cholesterol (LDL-C), total cholesterol (TC), and fasting plasma glucose. Notably, lipid and fasting glucose measurements were exclusively performed on participants who had fasted for a minimum of 8.5 h and a maximum of 24 h, thereby ensuring the validity of these assessments. Diabetes mellitus was defined as fasting blood glucose (FBG) ≥ 7.0 mmol/L, or a documented history of diabetes. Hypertension was defined as mean systolic blood pressure (SBP) ≥ 140 mmHg, mean diastolic blood pressure (DBP) ≥ 90 mmHg, and/or documented use of antihypertensive therapy (defined as affirmative responses to the question regarding current use of prescription medications for hypertension). Hyperlipidemia was defined as total cholesterol (TC) ≥ 5.2 mmol/L, triglycerides (TG) ≥ 1.7 mmol/L, low-density lipoprotein cholesterol (LDL-C) ≥ 3.4 mmol/L, high-density lipoprotein cholesterol (HDL-C) ≤ 1.3 mmol/L for females and ≤ 1.0 mmol/L for males, and/or documented use of lipid-lowering medications (defined as affirmative responses to the question regarding current use of prescription medications for lowering cholesterol).Affirmative responses to the question regarding current use of lipid-lowering medications were defined as receiving lipid-lowering therapy.

### Statistical analysis

The baseline characteristics of the study participants are presented as percentages for categorical variables, as the means with standard deviations for normally distributed variables, and as medians with interquartile ranges for nonnormally distributed variables. Both men and women were categorized into four groups based on the sex-specific quartile of the WWI. Demographic and clinical characteristics were compared among the four groups by ANOVA or the Kruskal‒Wallis test for continuous variables and the X^2^ test for categorical variables.

In the CHARLS cohort, disease history was ascertained through self-report, with incident disease onset dates documented throughout the follow-up period. As an initial exploratory analysis, Kaplan-Meier survival curves and log-rank tests (unadjusted for covariates) were first employed to evaluate the association between WWI and incident CKD. Cox proportional hazards models were subsequently fitted to estimate hazard ratios (HRs) and 95% confidence intervals (CIs) for the association between WWI quartiles and CKD incidence, with confounding control achieved through three hierarchically nested models: Model 1 adjusted for age; Model 2 further adjusted for BMI, smoking status, alcohol consumption, and history of chronic comorbidities (dyslipidemia, diabetes, and hypertension) in addition to Model 1; Model 3 further adjusted for uric acid, total cholesterol, HDL-C, and fasting glucose based on Model 2. The proportional hazards assumption for all Cox models was verified using Schoenfeld residuals.

To assess dose-response relationships, unrestricted cubic splines of WWI were fitted to estimate CKD risk continuously across the full exposure range. Restricted cubic splines (RCS) regression was then employed to characterize potential non-linear associations between WWI and CKD incidence. Effect modification was evaluated through subgroup analyses stratified by age (< 60 versus ≥ 60 years), alcohol consumption, smoking status, and history of hypertension, dyslipidemia, diabetes, and obesity. Interaction tests were conducted using likelihood ratio tests comparing models with and without product interaction terms. Statistical significance was determined at a two-sided alpha level of 0.05. All statistical analyses were performed using R software, with Cox regression and spline analyses implemented using the survival and rms packages, respectively. Additionally, sensitivity analysis was used to explore the robustness of our findings.

### Ethics statement

This study utilized publicly available data from the CHARLS, which received ethical approval from the Peking University Institutional Review Board (IRB approval No. IRB00001052-11015). All participants provided informed consent, and the study protocol was conducted in accordance with the Declaration of Helsinki principles.

## Results

### Baseline characteristics

Overall, 10,200 aged adults (4809 males and 5391 females) were included in the analysis, with an average age of 67.43 ± 9.55 years. The quartiles for WWI in males were as follows: Q1 (≤ 10.36), Q2 (10.37–10.80), Q3 (> 10.81–11.26), and Q4 (> 11.27), and the quartiles were as follows: Q1 (≤ 10.85), Q2 (10.86–11.42), Q3 (11.43–12.00), and Q4 (> 12.01) for females. Results demonstrated that, across both sexes, advancing WWI quartiles were associated with stepwise increases in age, BMI, waist circumference, body weight, UA, TC, LDL-C, and fasting plasma glucose, whereas HDL-C exhibited a declining trend (all *P* < 0.05). In the female subgroup, participants with higher WWI were more likely to reside in rural areas, present with obesity, and have hypertension, diabetes, or dyslipidemia; notably, smoking prevalence was also marginally elevated (*P* = 0.024). Similarly, among men, elevated WWI was significantly associated with rural residency, obesity, hypertension, diabetes, and dyslipidemia. Interestingly, smoking prevalence decreased across increasing WWI quartiles (*P* < 0.001), whereas alcohol consumption showed no significant variation (*P* = 0.086).(Supplement Tables 1 and 2). During the analysis of sex differences, age, education, smoking status, residential history, history of chronic diseases, hypertension status, diabetes status, BMI, UA levels, total cholesterol levels, HDL-C levels and LDL-C levels were significantly different (*P* < 0.05); only hyperlipidemia and blood glucose levels were not significantly different (*P* > 0.05) (Table 1).

### Survival curve analysis

**Fig. 2:**
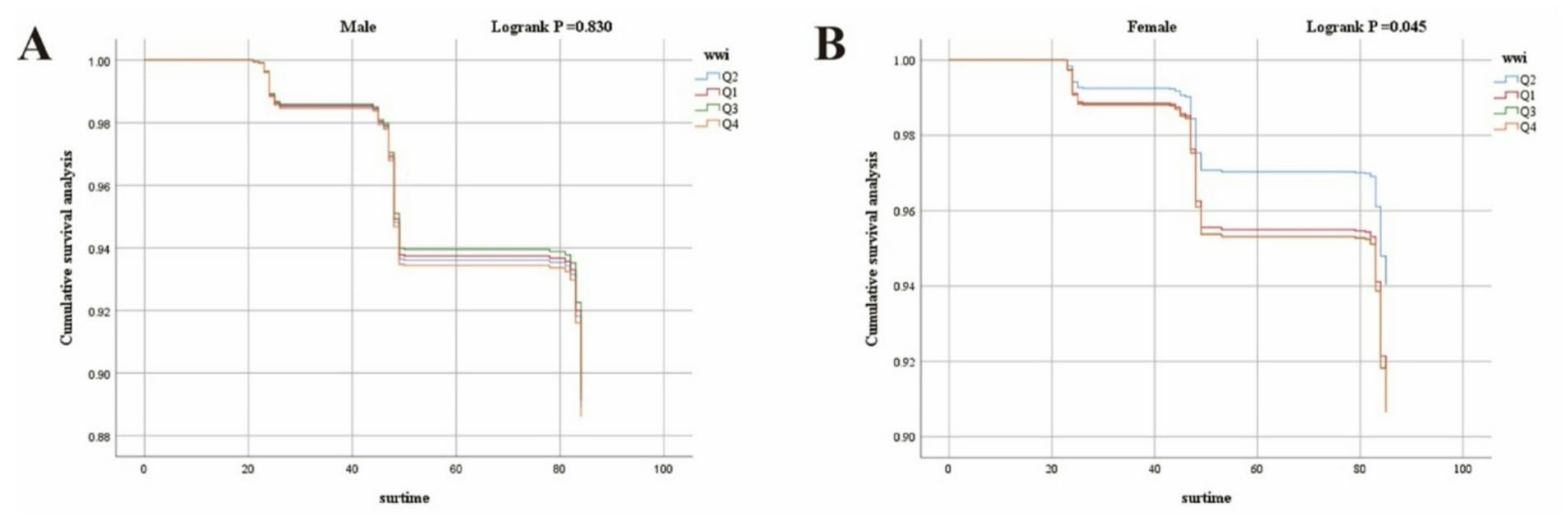
Kaplan–Meier survival analysis of CKD incidence according to WWI categories (crude model, unadjusted for covariates). Abbreviations: WWI, weight-adjusted waist circumference index.

Without adjustment for confounding factors, the log-rank test indicated no significant difference in survival rates across WWI quartiles in the male population (*P* = 0.830). However, for female without confounding factors, the log-rank test indicated a statistically significant difference in survival rates between WWI quartiles (*P* = 0.045) (Fig. 2).

### The relationship between the WWI and CKD via the Cox proportional hazards model

Using Cox proportional hazards models, we examined the association between WWI quartiles and the risk of incident CKD, with stepwise adjustment for confounding factors. The results showed that, in men, CKD risk across WWI groups was not statistically significant in any model. Point estimates hazard ratios (HR) were close to 1.0, with generally wide 95% confidence intervals (CIs) (e.g., in Model 3, Q1: HR = 1.19, 95% CI 0.82–1.71), indicating high uncertainty in the effect estimates and minimal change with covariate adjustment. In contrast, women exhibited a U-shaped association. Compared with the reference group (Q2), both the lowest (Q1) and highest (Q4) WWI groups showed significantly elevated CKD risk after full adjustment for age, lifestyle, comorbidities, and metabolic parameters (Model 3) (Q1: HR = 1.56, 95% CI 1.04–2.34; Q4: HR = 1.53, 95% CI 1.01–2.31). The Q3 group was also significantly associated (HR = 1.59, 95% CI 1.08–2.34). Although the 95% confidence intervals were relatively wide, they consistently excluded the null value of 1.0, suggesting a consistent direction of effect. Moreover, these estimates remained stable across models, supporting a consistent U-shaped relationship between WWI and CKD risk in women. Interaction analysis further revealed a statistically significant sex difference (*P* for interaction < 0.05), indicating that gender may modify the effect of WWI on CKD risk. This association persisted after controlling for potential confounders, supporting its independence (Table 2).

Based on nonrestrictive cubic splines, we found no nonlinear relationship between the WWI and CKD, regardless of sex (*P* > 0.05) (Supplementary Fig. 1).

### The relationship between the WWI and CKD according to subgroup

**Fig. 3:**
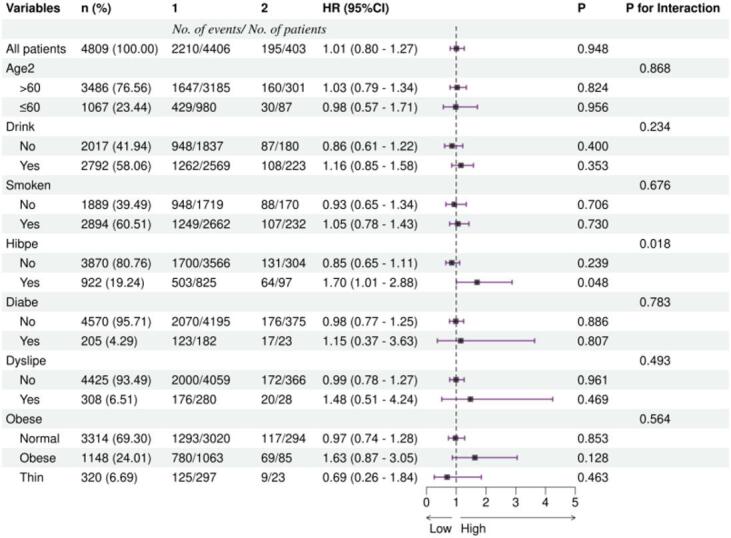
Subgroup analysis of the HR (95% CI) of the WWI for CKD in male populations. Abbreviations: Diabe, diabetes mellitus; Dyslipe, dyslipidemia; Hibpe, hypertension.

**Fig. 4:**
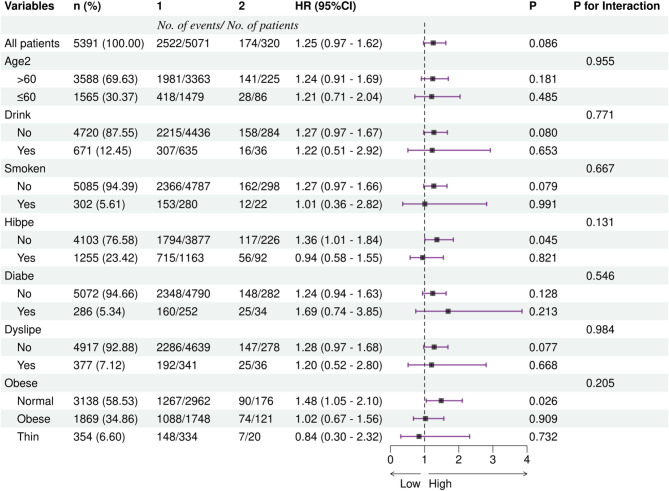
Subgroup analysis of the HR (95% CI) of the WWI for CKD in female populations. Abbreviations: Diabe, diabetes mellitus; Dyslipe, dyslipidemia; Hibpe, hypertension.

Further analysis will divide WWI into two categories, with median as the boundary, to analyze the relationship between WWI and CKD in different subgroups. Research findings, In the subgroup analysis, significant associations of higher WWI with risk of CKD were observed in Hypertension subgroups in male and a significant interaction between WWI groups and Hypertension on CKD was observed, However, no significant association between WWI and CKD was found in other subgroups of male participants(Fig. 3). In Female, research findings significant associations of higher WWI with risk of CKD were observed in no Hypertension subgroups, but significant associations of higher WWI with risk of CKD were observed. And we also found that significant associations of higher WWI with risk of CKD were observed in no normal obese subgroups, but significant associations of higher WWI with risk of CKD were observed, However, no significant association between WWI and CKD was found in other subgroups of female participants (Fig. 4).

### Predictive performance of different obesity indices for CKD in female populations

**Fig. 5:**
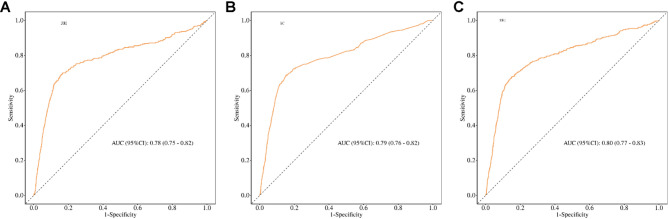
Predictive performance of different obesity indices for CKD in female populations. Abbreviations: BMI, body mass index; WC, waist circumference; WWI, weight-adjusted waist circumference index.

To further validate the predictive value of WWI for CKD, we performed ROC curve analyses for BMI, WC, and WWI. The results showed that the AUC value was 0.78 (95% CI 0.75–0.82) for BMI, 0.79 (95% CI 0.76–0.82) for WC, and 0.80 (95% CI 0.77–0.83) for WWI. Although the three AUC values were comparable, WWI was slightly higher. Furthermore, pairwise comparisons of the AUCs among the three indicators using DeLong’s test revealed statistically significant differences between WWI and BMI, as well as between WWI and WC (*p* < 0.05), suggesting that WWI has superior predictive ability for CKD compared to traditional obesity indices BMI and WC (Fig. 5).

### Sensitivity analysis

To assess the robustness of our findings, we conducted a sensitivity analysis on the common causes of CKD, including diabetes and hypertension. Consequently, the male WWI still had no statistically significant effect on the occurrence of CKD after excluding diabetic patients from the study. Among females, both Q3 and Q4 (vs. Q2 as reference) sustained positive associations with CKD risk, consistent with the primary analysis. Similarly, after excluding participants with hypertension, results remained materially unchanged, WWI was not significantly associated with CKD in men. While women in Q3 and Q4 (vs. Q2) demonstrated persistently elevated risks of incident CKD, consistent with the findings before exclusion (Table 3). These sensitivity analyses confirm the robustness of our results.

## Discussion

This prospective cohort study of 10,200 middle-aged and older Chinese adults demonstrated that elevated WWI was significantly associated with increased risk of incident CKD in women, even after rigorous adjustment for metabolic confounders including BMI, blood pressure, and glycemic status. Strikingly, this association was absent in men, highlighting a pronounced sexual dimorphism in the relationship between central adiposity patterns and renal outcomes. This sexual dimorphism may be partially attributable to distinct metabolic perturbations characteristic of postmenopausal women: the decline in estrogen levels precipitates a redistribution of adipose tissue with marked augmentation of visceral fat depots, while the hormonal “withdrawal effect” promotes adipocyte hypertrophy and compromises the lipid-buffering capacity of adipose tissue, thereby subjecting the kidneys to increased exposure of circulating free fatty acids and concomitant reduction of renoprotective adipokines such as adiponectin, which synergistically accelerate CKD pathogenesis.These findings provide novel longitudinal evidence supporting WWI as a sex-specific predictor of CKD development in the Chinese population.

CKD remains a substantial global health burden with limited effective prevention strategies, particularly in aging populations^19^. While obesity is recognized as a modifiable risk factor for CKD, traditional anthropometric indices such as BMI cannot distinguish between muscle and fat mass, potentially obscuring true metabolic risk^20,21^. Therefore, accumulating evidence suggests that visceral adiposity may be more critical than BMI per se in predicting adverse renal outcomes^4,22,23^. WWI, calculated by dividing waist circumference by the square root of body weight, offers a refined assessment of body composition by being positively correlated with fat mass and negatively with muscle mass^24^. Although previous cross-sectional studies have linked WWI with CKD prevalence^15,16^, longitudinal evidence regarding its predictive value for CKD onset, particularly concerning sex-specific patterns, remains scarce. The present ROC curve analysis further demonstrates that WWI exhibits superior predictive performance for CKD compared with traditional obesity indices (BMI and WC) in women. Collectively, this study confirms that WWI can independently predict future CKD risk in women beyond conventional metabolic risk factors, thereby filling this critical knowledge gap.

Obesity and CKD are both recognized as major public health problems. Obesity can trigger insulin resistance, chronic inflammation, and activation of the sympathetic nervous system. These factors, along with increased complications such as hypertension, diabetes, and vascular sclerosis and the mechanical stress caused by fat accumulation, are believed to be promising targets for understanding the development of both WWI and CKD^23,25^. A prospective study of CKD risk at 7 years of follow-up revealed that weight loss surgery significantly improved the risk of CKD in patients^26^. Therefore, body weight control and reducing the level of visceral fat may be effective approaches for reducing the impact of obesity on the kidneys.

Hypertension constitutes a critical confounder in the male cohort, with its complexity stemming from sex-specific differences in physiology, pathophysiology, and lifestyle factors. Pathogenetic mechanisms and disease progression patterns of hypertension differ markedly between sexes. Specifically, elevated androgen levels, enhanced sympathetic nervous activity, and differential regulation of the renin-angiotensin-aldosterone system (RAAS) may synergistically drive hypertension development and renal injury progression^27^. Moreover, visceral fat accumulation is notably more pronounced in males^28,29^. Notably, visceral adipose tissue—specifically perirenal adipose tissue (PRAT)—has been implicated in the pathogenesis of both hypertension and CKD. PRAT contributes to renal impairment through mechanical compression, pro-inflammatory cytokine secretion, and RAAS activation^30^. Accordingly, hypertension represents not merely an independent risk factor for CKD, but may also engage in complex interactions with WWI or serve as a mediating variable.

This study possesses several methodological strengths. The prospective design with an 8.0-year follow-up establishes clear temporal precedence of exposure over outcome. The large, nationally representative sample from CHARLS enhances generalizability to Chinese middle-aged and older populations. Furthermore, comprehensive adjustment for metabolic confounders and multiple sensitivity analyses bolster the robustness of our findings.

This study has several limitations. First, baseline eGFR and UACR were not routinely collected in the parent cohort design. Although we mitigated this by excluding participants with prevalent CKD and adjusted for multiple established risk factors closely associated with kidney function, including age, sex, hypertension, diabetes, and dyslipidemia., residual confounding may persist. Second, we examined only baseline WWI values without accounting for temporal trajectories during the 8-year follow-up, which may limit precision of exposure-outcome associations. Third, CKD diagnosis relied partially on self-report with only 10% awareness rate, risking misclassification bias. The CKD-EPI equation, based on creatinine (a muscle metabolism byproduct), may overestimate eGFR in high-WWI individuals (indicating greater visceral adiposity but potentially lower muscle mass), introducing “composition bias.” Future studies should validate these findings in cohorts with comprehensive renal biomarkers and repeated WWI measurements.

**Table 1:** Baseline characteristics of the study participants according to sex.

Characteristics	sex		t/x^2^	*p*
	Male (*n* = 4809)	Female (*n* = 5391)		
No. of subjects	4809	5391		
Age				
Mean ± SD	68.08 ± 9.17	66.86 ± 9.83	− 6.307	0.000**
Education level, n (%)				
Junior middle school	5029(93.29)	4116(85.59)	162.774	0.000**
High school	318(5.90)	598(12.44)		
College or above	44(0.82)	95(1.98)		
Place of residence, n (%)				
Urban	3922(81.56)	4556(84.51)	18.092	0.001**
Rural	885(18.40)	829(15.38)		
Drinking, n (%)				
Yes	2792(58.06)	671(12.45)	2358.018	0.001**
Smoking, n (%)				
Yes	2894(60.18)	302(5.60)	3561.31	0.001**
Chronic disease history, n (%)				
Yes	922(19.17)	1255(23.28)	29.754	0.001**
Hypertension, n (%)				
Yes	205(4.26)	286(5.31)	6.326	0.042*
Diabetes, n (%)				
Yes	205(4.26)	286(5.31)	6.326	0.042*
Dyslipidemia, n (%)				
Yes	308(6.40)	377(6.99)	2.21	0.331
BMI				
Mean ± SD	22.76 ± 3.26	23.76 ± 3.73	14.427	0.000**
UA				
M (IQR)	4.79(4.1,5.7)	3.860(3.3,4.6)	− 34.414	0.000**
Total cholesterol				
M (IQR)	185.18(162.8,208.4)	194.46(170.5,220.8)	− 10.831	0.000**
HDL-C				
M (IQR)	48.71(39.4,59.5)	50.64(41.8,60.7)	− 5.015	0.000**
LDL-C				
M (IQR)	110.57(90.5,131.4)	117.53(95.5,141.9)	− 9.502	0.000**
Blood glucose (mg/dl)				
M (IQR)	102.78(94.1,113.8)	101.88(94.3,112.3)	− 1.475	0.143

Mean ± SD for continuous variables: The *p* value was calculated by the weighted linear regression model.

(%) For categorical variables, the *p* value was calculated by the weighted chi-square test, * *P* < 0.05 ** *P* < 0.001.

BMI, body mass index; CKD, chronic kidney disease; HDL-C, high-density lipoprotein cholesterol; LDL-C, low-density lipoprotein cholesterol; TC, total cholesterol; UA, uric acid; WWI, weight-adjusted waist circumference index.

**Table 2:** Association between the WWI and CKD in male and female subjects.

Gender	WWI	Rate/1000 p_years	Case, (*n*%)	CKD		
				Model 1	Model 2	Model 3
				HR (95% CI)	HR (95% CI)	HR (95% CI)
	Total		403(8.38)			
Male	≦ 10.36	14.04	105(8.80)	0.99 (0.74–1.31)	1.01 (0.75–1.37)	1.19 (0.82–1.71)
	10.37–10.80	13.75	102(8.51)	Ref	Ref	Ref
	10.81–11.26	12.72	95(7.85)	0.99 (0.75–1.31)	1.11 (0.82–1.52)	1.10 (0.78–1.54)
	≧ 11.27	13.92	101(8.37)	0.92 (0.69–1.22)	1.05 (0.79–1.40)	1.08 (0.76–1.54)
Female	Total		320(5.94)			
	≦ 10.85	9.22	78(5.86)	1.29 (0.92–1.80)	1.42 (1.01–2.00)	1.56 (1.04–2.34)
	10.86–11.42	7.51	66(4.89)	Ref	Ref	Ref
	11.43-12.00	9.63	84(6.16)	1.30 (0.93–1.81)	1.31 (0.93–1.85)	1.59 (1.08–2.34)
	≧ 12.01	11.09	92(6.83)	1.57 (1.12–2.20)	1.50 (1.05–2.13)	1.53 (1.01–2.31)

Male: Q1 ( ≦ 10.36), Q2 (10.37–10.80), Q3 (10.81–11.26), and Q4 ( ≧ 11.27); Female: Q1 ( ≦ 10.85), Q2 (10.86–11.42), Q3 (11.43-12.00), and Q4 ( ≧ 12.01).

Model 1 was adjusted for age.

Model 2 was further adjusted for BMI, smoking status, drinking status, and chronic diseases (dyslipidemia, diabetes, hypertension) based on Model 1.

Model 3 was further adjusted for UA, total cholesterol, HDL, and blood glucose based on Model 2.

CKD, chronic kidney disease; WWI, weight-adjusted waist circumference index.

**Table 3:** Sensitivity analysis of the common causes of CKD.

Variables		Exclude people with diabetes	Exclude people with hypertension
		HR (95%CI)	HR (95%CI)
	WWI		
Male	≦ 10.36	1.39 (0.91 ,2.10)	1.37(0.86,2.19)
	10.86–11.42	Ref	Ref
	11.43-12.00	0.92(0.65,1.30)	0.86(0.60,1.25)
	≧ 12.01	1.04(0.74,1.46)	0.73(0.49,1.09)
Female	10.86–11.42	Ref	Ref
	10.37–10.80	Ref	Ref
	10.81–11.26	1.46 (1.08 ,2.18)	1.76(1.12,2.74)
	≧ 11.27	1.48 (1.09 ,2.23)	1.75(1.08,2.83)

A sensitivity analysis of the common causes of CKD, including diabetes and hypertension, was performed to test the robustness of the results.

CKD, chronic kidney disease; WWI, weight-adjusted waist circumference index.

## Conclusion

In conclusion, this study provides novel evidence that elevated WWI is independently associated with incident CKD risk in Chinese women but not men. These results underscore the importance of assessing body composition quality—specifically central adiposity relative to body size—in CKD prevention strategies for female populations. Future research should validate these sex-specific findings in diverse cohorts and investigate whether targeted interventions optimizing body composition can reduce CKD incidence, particularly among in middle-aged and older Chinese women.

## Electronic Supplementary Material

Below is the link to the electronic supplementary material.

Supplementary Material 1

## Data Availability

The datasets generated during and/or analyzed during the current study are available in the CHARLS repository, http://charls.pku.edu.cn.
