# Supplementary material for: Association between a novel obesity index WWI and chronic kidney disease in a Chinese middle-aged and older population: a national prospective cohort study
Source: Sci Rep. 2026 Apr 18;16:18090. doi: 10.1038/s41598-026-48320-w (PMC13254146; doi:10.1038/s41598-026-48320-w)
Supplement: Supplementary file 1 — Supplementary Material 1 [file 41598_2026_48320_MOESM1_ESM.docx]

1 Supplement Table

Supplement Table 1 Baseline characteristics of female participants stratified by WWI quartiles

| **Variables** |  | **WWI(%)** | | | | **Total (n = 5391)** | **χ2** | **p** |
| --- | --- | --- | --- | --- | --- | --- | --- | --- |
|  |  | **Q1** | **Q2** | **Q3** | **Q4** |  |  |  |
| register | Rural | 1077(80.98) | 1147(85.15) | 1165(85.54) | 1167(86.70) | 4556(84.61) | 19.195 | 0.000** |
|  | Urban | 253(19.02) | 200(14.85) | 197(14.46) | 179(13.30) | 829(15.39) |  |  |
| drink | No | 1157(86.93) | 1178(87.26) | 1202(88.19) | 1183(87.82) | 4720(87.55) | 1.181 | 0.758 |
|  | Yes | 174(13.07) | 172(12.74) | 161(11.81) | 164(12.18) | 671(12.45) |  |  |
| smoken | No | 1249(93.91) | 1294(95.85) | 1286(94.56) | 1256(93.24) | 5085(94.39) | 9.446 | 0.024* |
|  | Yes | 81(6.09) | 56(4.15) | 74(5.44) | 91(6.76) | 302(5.61) |  |  |
| Obese | Normal | 930(70.08) | 840(62.41) | 683(50.48) | 685(51.31) | 3138(58.53) | 227.563 | 0.000** |
|  | Obese | 271(20.42) | 433(32.17) | 607(44.86) | 558(41.80) | 1869(34.86) |  |  |
|  | Thin | 126(9.50) | 73(5.42) | 63(4.66) | 92(6.89) | 354(6.60) |  |  |
| hibpe | No | 1124(85.02) | 1056(78.81) | 1024(75.52) | 899(67.09) | 4103(76.58) | 124.381 | 0.000** |
|  | Yes | 198(14.98) | 284(21.19) | 332(24.48) | 441(32.91) | 1255(23.42) |  |  |
| diabe | No | 1291(97.21) | 1271(95.21) | 1257(92.70) | 1253(93.58) | 5072(94.66) | 31.354 | 0.000** |
|  | Yes | 37(2.79) | 64(4.79) | 99(7.30) | 86(6.42) | 286(5.34) |  |  |
| dyslipe | No | 1248(95.27) | 1223(92.58) | 1234(91.75) | 1212(91.96) | 4917(92.88) | 15.77 | 0.001** |
|  | Yes | 62(4.73) | 98(7.42) | 111(8.25) | 106(8.04) | 377(7.12) |  |  |
| age, | Mean ± SD | 62.09 ± 7.96 | 64.70 ± 8.53 | 67.20 ± 9.00 | 73.50 ± 9.97 | 66.86 ± 9.83 | F=384.75 | **<.001** |
| BMI, | Mean ± SD | 22.63 ± 3.46 | 23.61 ± 3.43 | 24.47 ± 3.69 | 24.34 ± 4.04 | 23.76 ± 3.73 | F=71.18 | **<.001** |
| wasit, | Mean ± SD | 76.35 ± 7.49 | 83.13 ± 7.52 | 87.92 ± 7.99 | 92.66 ± 9.09 | 85.05 ± 10.05 | F=1002.45 | **<.001** |
| weight | Mean ± SD | 54.85 ± 9.53 | 56.09 ± 10.01 | 57.00 ± 10.17 | 54.48 ± 10.98 | 55.61 ± 10.23 | F=17.48 | **<.001** |
| UA | M (Q₁, Q₃) | 3.61 (3.10,4.29) | 3.78 (3.22,4.48) | 3.94 (3.34,4.63) | 4.09 (3.45,4.77) | 3.86 (3.26, 4.56) | χ²=100.47# | **<.001** |
| Cho | ,M (Q₁, Q₃) | 186.34 (164.30,208.38) | 193.69 (172.04,218.04) | 196.20 (171.65,222.97) | 201.03 (175.90,229.16) | 194.46 (170.49, 220.75) | χ²=84.05# | **<.001** |
| Hdl | ,M (Q₁, Q₃) | 54.12 (44.85,64.18) | 51.03 (42.14,59.92) | 49.10 (40.98,59.15) | 48.33 (40.01,58.76) | 50.64 (41.75, 60.70) | χ²=75.60# | **<.001** |
| Ldl | M (Q₁, Q₃) | 111.34 (91.24,131.06) | 119.46 (97.42,142.66) | 119.46 (95.20,145.36) | 120.62 (99.55,147.68) | 117.53 (95.49, 141.88) | χ²=61.21# | **<.001** |
| Glu | ,M (Q₁, Q₃) | 99.18 (92.52,107.64) | 101.52 (93.96,111.24) | 102.60 (94.32,114.66) | 104.04 (96.30,116.41) | 101.88 (94.32, 112.32) | χ²=91.61# | **<.001** |

Note: Female: Q1 (≦10.85), Q2 (10.86-11.42), Q3 (11.43-12.00), and Q4 (≧12.01).

F: ANOVA, #: Kruskal-waills test， SD: standard deviation, M: Median, Q₁: 1st Quartile, Q₃: 3st Quartile

Supplement Table 2 Baseline characteristics of male participants stratified by WWI quartiles

| Variables |  | WWI(%) | | | | **Total (n = 4809)** | χ2 | p |
| --- | --- | --- | --- | --- | --- | --- | --- | --- |
|  |  | Q1 | Q2 | Q3 | Q4 |  |  |  |
| register | Rural | 1019(85.41) | 998(83.24) | 961(79.55) | 944(78.21) | 3922(81.59) | 26.297 | 0.000** |
|  | Urban | 174(14.59) | 201(16.76) | 247(20.45) | 263(21.79) | 885(18.41) |  |  |
| drink | No | 496(41.58) | 482(40.20) | 496(40.99) | 543(44.99) | 2017(41.94) | 6.606 | 0.086 |
|  | Yes | 697(58.42) | 717(59.80) | 714(59.01) | 664(55.01) | 2792(58.06) |  |  |
| smoken | No | 411(34.71) | 440(36.82) | 511(42.34) | 527(44.03) | 1889(39.49) | 29.274 | 0.000** |
|  | Yes | 773(65.29) | 755(63.18) | 696(57.66) | 670(55.97) | 2894(60.51) |  |  |
| Obese | Normal | 970(81.99) | 923(77.30) | 774(64.13) | 647(54.01) | 3314(69.30) | 404.397 | 0.000** |
|  | Obese | 96(8.11) | 203(17.00) | 378(31.32) | 471(39.32) | 1148(24.01) |  |  |
|  | Thin | 117(9.89) | 68(5.70) | 55(4.56) | 80(6.68) | 320(6.69) |  |  |
| hibpe | No | 1037(87.36) | 990(82.85) | 960(79.54) | 883(73.40) | 3870(80.76) | 79.755 | 0.000** |
|  | Yes | 150(12.64) | 205(17.15) | 247(20.46) | 320(26.60) | 922(19.24) |  |  |
| diabe | No | 1162(97.98) | 1152(96.73) | 1145(95.26) | 1111(92.89) | 4570(95.71) | 41.512 | 0.000** |
|  | Yes | 24(2.02) | 39(3.27) | 57(4.74) | 85(7.11) | 205(4.29) |  |  |
| dyslipe | No | 1133(96.43) | 1108(94.06) | 1107(93.03) | 1077(90.50) | 4425(93.49) | 35.126 | 0.000** |
|  | Yes | 42(3.57) | 70(5.94) | 83(6.97) | 113(9.50) | 308(6.51) |  |  |
|  |  |  |  |  |  |  |  |  |
| age | Mean ± SD | 66.14 ± 8.08 | 66.99 ± 8.55 | 67.91 ± 9.19 | 71.28 ± 9.91 | 68.08 ± 9.17 | F=71.58 | <.001 |
| BMI | Mean ± SD | 21.26 ± 2.46 | 22.38 ± 2.70 | 23.45 ± 3.24 | 23.93 ± 3.81 | 22.76 ± 3.26 | F=175.61 | <.001 |
| wasit | Mean ± SD | 76.06 ± 6.21 | 82.35 ± 6.59 | 87.17 ± 7.89 | 92.39 ± 9.20 | 84.52 ± 9.67 | F=1013.89 | <.001 |
| weight | Mean ± SD | 58.72 ± 8.56 | 60.88 ± 9.55 | 63.09 ± 11.31 | 62.96 ± 12.22 | 61.42 ± 10.66 | F=46.09 | <.001 |
| UA | M (Q₁, Q₃) | 4.62 (3.95,5.39) | 4.76 (4.03,5.54) | 4.92 (4.16,5.82) | 5.00 (4.21,5.93) | 4.79 (4.07, 5.66) | χ²=56.40# | <.001 |
| Cho | M (Q₁, Q₃) | 179.38 (158.51,204.12) | 184.02 (163.73,208.18) | 188.66 (165.85,212.63) | 188.27 (164.30,210.31) | 185.18 (162.76, 208.38) | χ²=30.52# | <.001 |
| Hdl | M (Q₁, Q₃) | 51.80 (42.91,61.47) | 49.87 (40.59,62.24) | 47.94 (38.66,57.60) | 45.62 (37.50,56.06) | 48.71 (39.43, 59.54) | χ²=83.03# | <.001 |
| Ldl | M (Q₁, Q₃) | 106.70 (89.30,126.03) | 109.79 (91.24,131.44) | 112.11 (90.08,134.54) | 112.50 (90.46,134.15) | 110.57 (90.46, 131.44) | χ²=12.86# | 0.005 |
| Glu | M (Q₁, Q₃) | 99.90 (91.98,108.72) | 102.06 (94.23,111.78) | 104.40 (95.40,115.38) | 104.40 (95.58,117.86) | 102.78 (94.14, 113.76) | χ²=60.06# | <.001 |

Note: Male: Q1 (≦10.36), Q2 (10.37-10.80), Q3 (10.81-11.26), and Q4 (≧11.27).

F: ANOVA, #: Kruskal-waills test， SD: standard deviation, M: Median, Q₁: 1st Quartile, Q₃: 3st Quartile

2 Supplement Fig


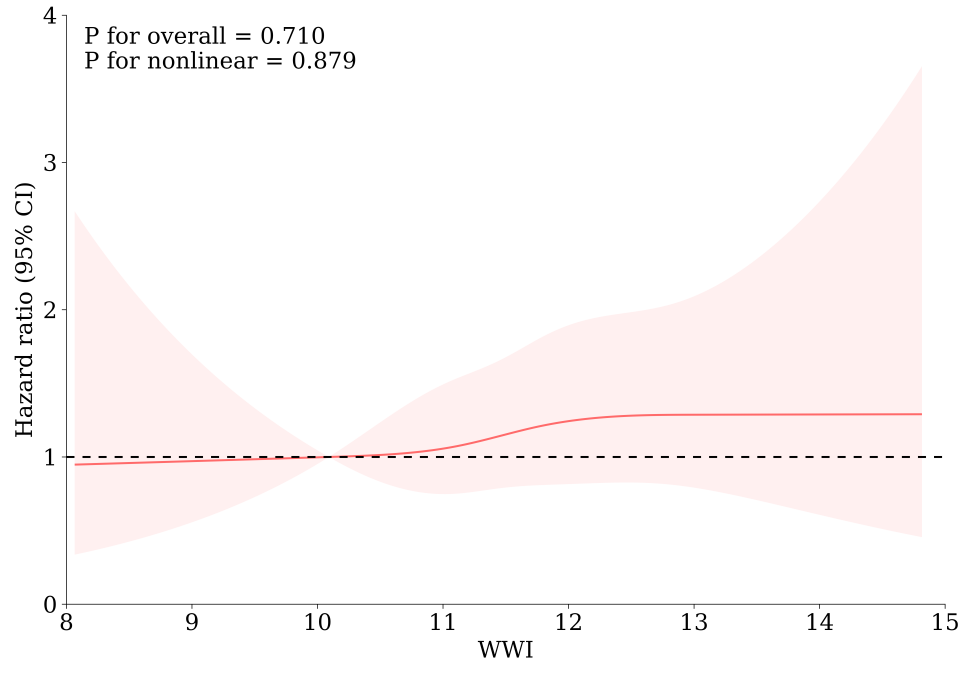

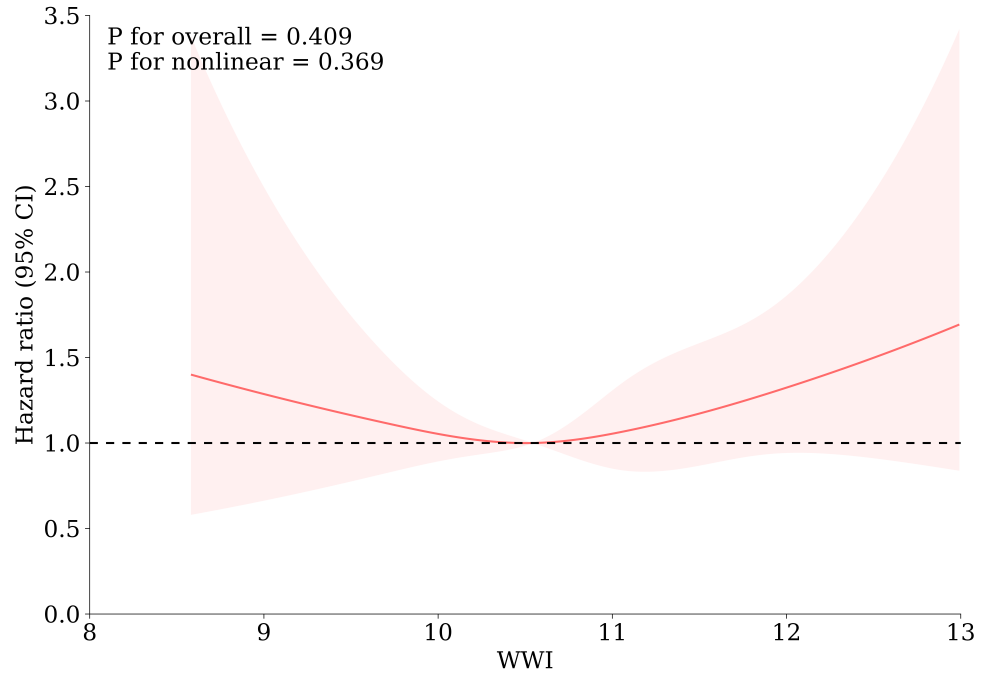


1. Male （2）Female

Supplement Fig1. Analyzing the relationship between WWI and CKD using non restrictive cubic splines
